# Birth weight differences between those offered financial voucher incentives for verified smoking cessation and control participants enrolled in the Cessation in Pregnancy Incentives Trial (CPIT), employing an intuitive approach and a Complier Average Causal Effects (CACE) analysis

**DOI:** 10.1186/s13063-017-2053-x

**Published:** 2017-07-20

**Authors:** Alex McConnachie, Caroline Haig, Lesley Sinclair, Linda Bauld, David M. Tappin

**Affiliations:** 1 Robertson Centre, Level 11, Boyd Orr Building, University of Glasgow, Glasgow, G12 8QQ UK; 2 Institute for Social Marketing, Centre for Tobacco and Alcohol Studies, Faculty of Health Sciences & Sport, University of Stirling, Stirling, FK9 4LA UK; 3 Section of Child Health, School of Medicine, Glasgow University, Scottish Cot Death Trust, 5th floor, West Glasgow Ambulatory Care Hospital, Yorkhill, Glasgow, G3 8SJ UK

**Keywords:** Treatment effectiveness, Birth weight, Smoking cessation, Pregnancy

## Abstract

**Background:**

The Cessation in Pregnancy Incentives Trial (CPIT), which offered financial incentives for smoking cessation during pregnancy showed a clinically and statistically significant improvement in cessation. However, infant birth weight was not seen to be affected. This study re-examines birth weight using an intuitive and a complier average causal effects (CACE) method to uncover important information missed by intention-to-treat analysis.

**Methods:**

CPIT offered financial incentives up to £400 to pregnant smokers to quit. With incentives, 68 women (23.1%) were confirmed non-smokers at primary outcome, compared to 25 (8.7%) without incentives, a difference of 14.3% (Fisher test, *p* < 0.0001). For this analysis, randomised groups were split into three theoretical sub-groups: independent quitters - quit without incentives, hardened smokers - could not quit even with incentives and potential quitters - required the addition of financial incentives to quit. Viewed in this way, the overall birth weight gain with incentives is attributable only to potential quitters. We compared an intuitive approach to a CACE analysis.

**Results:**

Mean birth weight of potential quitters in the incentives intervention group (who therefore quit) was 3338 g compared with potential quitters in the control group (who did not quit) 3193 g. The difference attributable to incentives, was 3338 – 3193 = 145 g (95% CI −617, +803). The mean difference in birth weight between the intervention and control groups was 21 g, and the difference in the proportion who managed to quit was 14.3%. Since the intervention consisted of the offer of incentives to quit smoking, the intervention was received by all women in the intervention group. However, “compliance” was successfully quitting with incentives, and the CACE analysis yielded an identical result, causal birth weight increase 21 g ÷ 0.143 = 145 g.

**Conclusions:**

Policy makers have great difficulty giving pregnant women money to stop smoking. This study indicates that a small clinically insignificant improvement in average birth weight is likely to hide an important clinically significant increase in infants born to pregnant smokers who want to stop but cannot achieve smoking cessation without the addition of financial voucher incentives.

**Trial Registration:**

ISRCTN Registry, ISRCTN87508788. Registered on 1 September 2011.

**Electronic supplementary material:**

The online version of this article (doi:10.1186/s13063-017-2053-x) contains supplementary material, which is available to authorized users.

## Background

Healthy birth weight is important for both early and long-term infant and child health as well as adult health. Healthcare costs are increased throughout life for low birth-weight babies. Babies born smaller develop more problems in the first month of life, requiring care in level 3 and level 4 neonatal intensive care units [1]. Small babies are more likely to die suddenly and unexpectedly in the first year of life [2]. They will also be less successful at attaining appropriate academic grades at school [3]. Infants who are small for gestational age are more likely to develop high blood pressure and other cardiovascular problems in later life [4, 5]. Babies born to smokers are more likely to develop obesity as they grow up and will generally be of lower birth weight even after adjusting for gestation [6].

Healthcare costs associated with lower birth weight are substantial and are at least partially avoidable if women do not smoke during pregnancy [7]. Smoking during pregnancy, as a risk factor on its own, will result in a baby born 160 g smaller to women who continue to smoke up to nine cigarettes per day and 230 g smaller to those who continue to smoke more than nine cigarettes per day) [8].

Interventions to help women to reduce or stop smoking during pregnancy, whilst of varying efficacy, have led to an overall moderate but statistically significant increase of 41 g in mean birth weight [9]. Financial incentive intervention studies carried out by one research group in the USA reported high efficacy (34.1% cessation) in the intervention group offered incentives contingent on biochemically validated cessation. This compared with 7.4% cessation in those offered incentives not contingent on cessation. Mean birth weight was 202 g greater in the intervention group [10]. This difference was highly statistically and clinically significant.

The Cessation in Pregnancy Incentives Trial (CPIT) [11] (Additional files 1 and 2) was a large single-centre trial with moderate to high efficacy, with a 23.5% quit rate in the group offered financial incentives for biochemically validated cessation, and 8.6% in the control group not offered incentives. This difference in validated cessation towards the end of pregnancy was highly significant. However, mean birth weight was only 21 g greater in the incentives group and this result was not statistically significant (*p* = 0.67).

This paper looks again at birth weight data from the CPIT and puts forward an alternative analysis. It aims to show the real effect on birth weight in women who would not quit without financial incentives but who manage to quit when financial incentives are added to routine cessation services. This intuitive approach is compared with a Complier Average Causal Effects (CACE) analysis.

## Methods

The CPIT was a phase II single-centre efficacy trial that offered up to £400 of financial incentives to self-reported pregnant smokers to engage with smoking cessation services and quit smoking near the end of pregnancy. Enrolment of participants included women who were representative of all self-reported pregnant smokers identified routinely at maternity booking in the National Health Service (NHS) Greater Glasgow and Clyde Health Board area [12]. The primary outcome was cotinine-validated self-report of smoking cessation collected between 34 and 38 weeks gestation corroborated by saliva (or urine) cotinine estimation. Excluding participants who had multiple births (n = 5), 607 women were randomised in the CPIT (304 in the incentives, 303 in the control group). Of these, the birth weight of their baby was not available for nine women in the incentives group and 16 in the control group, leaving 582 mother/baby pairs for analysis.

In the incentives group, 68 women (23.1%) were confirmed non-smokers at 34–38 weeks gestation compared to 25 women (8.7%) in the control group, a difference of 14.3% (Fisher test, *p* < 0.0001). The mean birth weight of women who stopped smoking was 3473 g, compared to 3065 g in women who continued to smoke, a difference of 408 g (two-sample *t* test, *p* < 0.0001). Given these differences, it might be expected that the difference in mean birth weight between randomised groups would be 0.143 × 408 g, or 59 g. In fact, the difference in birth weight between randomised groups was only 21 g (two-sample *t* test, *p* = 0.67).

To examine why the CPIT intention-to-treat (ITT) analysis, unlike other trials [10], showed no significant increase in birth weight despite reporting highly significant differences in both the quit rate in those offered incentives and those not [11] and the birth weight of babies born to those who quit smoking and those who did not, we intuitively examined birth weights by group (incentives, no incentives, quitters, non-quitters). We compared our results with those of CACE analysis.

## Statistical analyses

Statistical analyses were performed with R for Windows v3.2.4 [13] and SAS for Windows v9.3 [14]. To obtain the confidence interval for the difference in birth weights, we used a bootstrapping approach; 10,000 replicated datasets were drawn with replacement from the original dataset, and for each replicate, estimates were obtained for the mean birth weight for various subgroups of women, under the assumptions of receiving the intervention or not. Mean values in each subgroup and differences between groups are reported with 95% bias-corrected and accelerated confidence intervals.

## Results

### Birth weight of babies by group allocation and primary outcome (cotinine-validated self-report of smoking cessation at 34–38 weeks gestation near the end of pregnancy)

**Fig. 1:**
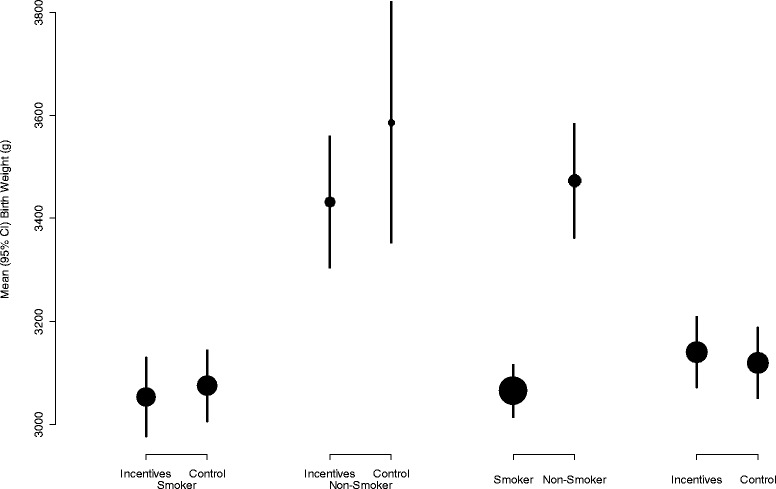
Mean (95% CI) birth weight (in g) in relation to the randomised group, and smoking status measured as the primary outcome. Size of *circles* is in proportion to the number of babies

Table 1 and Fig. 1 show the birth weights of babies born to women in the trial, broken down by randomised group and cotinine-validated smoking cessation near the end of pregnancy. As can be seen, babies of women in the incentives group who continued to smoke had a birth weight 22 g less than continuing smokers in the control group. Similarly, women in the incentives group who managed to quit had babies with a mean birth weight 154 g less than control group women who stopped smoking. Whilst neither of these differences was statistically significant, it is noticeable in both scenarios that birth weights in the incentives group were lower than in the control group. Nevertheless, since more women in the incentives group were able to stop smoking, the net effect was that babies born to women in the incentives group were on average slightly bigger (21 g).

**Table 1:** Birthweight (g) by randomised group and smoking status at end of pregnancy (primary outcome)

		Primary outcome		All
		Smoker	Non-smoker	
Randomised group	Control	N = 262 3075 (569)	N = 25 3586 (566)	N = 287 3120 (586)
	Incentives	N = 227 3053 (588)	N = 68 3432 (527)	N = 295 3141 (595)
All		N = 489 3065 (577)	N = 93 3473 (539)	N = 582 3130 (590)

Results are presented as number of observations (N) and mean (standard deviation); 25 birth weights were missing, as they were not available from routinely collected data

### Intuitive approach to assess the effect of incentives hidden by ITT analysis

Those women in the incentives group who continued to smoke were doing so despite the offer of incentives to quit. In that respect, they can be seen as a group of “hardened smokers”, who could not be encouraged to quit with the incentives on offer. It is not unreasonable to think that these women may lead the least healthy lifestyles of those in the trial. These are the women who had the smallest babies.

Similarly, those in the control group who managed to quit, did so despite not being offered any additional incentive, beyond the health benefits of stopping smoking. These women can be viewed as “independent quitters”, i.e. the least entrenched in their habit, and most able to quit. It may well be the case that these women adopted other healthy lifestyle choices during their pregnancies. These women had the largest babies.

Following this logic, the control group women who continued to smoke can be seen to be made up of two subgroups: the hardened smokers as aforementioned, plus women who might have been able to quit, if they had been offered incentives. By the same token, women in the incentives group who quit smoking likely includes some independent quitters, plus women who managed to stop smoking as a result of being offered incentives (and who might not have quit had they not been offered any incentives).

Therefore, both the control group smokers, and the incentives group quitters, include women in a third subgroup who, when offered incentives are able to stop smoking, but without incentives, continue to smoke. This group could be viewed as “potential quitters”, who probably tend to make (or are more able to make) healthier lifestyle choices than the hardened smokers, but not to the same extent as the independent quitters.

If we define the probabilities of being a hardened smoker, an independent quitter or a potential quitter as P_HS_, P_IQ_ and P_PQ_, respectively, then from the proportion of the incentives group who continued to smoke, we can estimate P_HS_ as 0.769 (76.9%); from the proportion of the control group who managed to quit, we can estimate P_IQ_ as 0.087 (8.7%). Therefore, we can estimate the proportion of potential quitters, P_PQ_, as 1 − 0.769 − 0.087 = 0.143 (14.3%). This is exactly the same percentage as the difference between the incentives group and the control group who managed to quit.

The mean birth weight for a baby of a hardened smoker, W_HS_, can be estimated from the mean birth weight of babies born to women in the incentives group who continued to smoke, i.e. 3053 g. The mean birth weight for a baby of an independent quitter, W_IQ_, can be estimated using the mean birth weight of babies born to women in the control group, who managed to quit, i.e. 3586 g.

What we are interested in is the mean birth weight of babies born to potential quitters who are either offered incentives and manage to quit (W_PQQ_), or who are not offered incentives and therefore continue to smoke (W_PQS_). The difference between these two values, W_PQQ_ − W_PQS_, can be seen as the gain in birth weight attributable to the offer of incentives, for babies born to women who are potential quitters. However, we do not have any direct means of estimating these two mean birth weights.

The mean birth weight of babies born to women in the incentives group who managed to quit (W_1_) can be thought of as a weighted mean of the birth weight of babies born to the independent quitters (W_IQ_) and the potential quitters who were able to quit (W_PQQ_) within this group:

$$ {\mathrm{W}}_1 = \left(\ {\mathrm{P}}_{\mathrm{IQ}}\times {\mathrm{W}}_{\mathrm{IQ}} + {\mathrm{P}}_{\mathrm{P}\mathrm{Q}}\times {\mathrm{W}}_{\mathrm{P}\mathrm{Q}\mathrm{Q}}\right) \div \left({\mathrm{P}}_{\mathrm{IQ}} + {\mathrm{P}}_{\mathrm{P}\mathrm{Q}}\right), $$

so that

$$ {\mathrm{W}}_{\mathrm{P}\mathrm{Q}\mathrm{Q}} = \left(\ {\mathrm{W}}_1\times \kern0.5em \left(\ {\mathrm{P}}_{\mathrm{IQ}} + {\mathrm{P}}_{\mathrm{P}\mathrm{Q}}\right)\ \hbox{-}\ {\mathrm{P}}_{\mathrm{IQ}}\times {\mathrm{W}}_{\mathrm{IQ}}\right) \div {\mathrm{P}}_{\mathrm{P}\mathrm{Q}} = 3338\mathrm{g}. $$

Similarly, the mean birth weight of babies born to women in the control group who continued to smoke (W_0_) can be seen as a weighted mean of the birth weight of babies born to the hardened smokers (W_HS_) and the potential quitters who were not able to quit (W_PQS_):

$$ {\mathrm{W}}_0 = \left(\ {\mathrm{P}}_{\mathrm{HS}}\times {\mathrm{W}}_{\mathrm{HS}} + {\mathrm{P}}_{\mathrm{P}\mathrm{Q}}\times {\mathrm{W}}_{\mathrm{P}\mathrm{Q}\mathrm{S}}\right) \div \left(\ {\mathrm{P}}_{\mathrm{HS}} + {\mathrm{P}}_{\mathrm{P}\mathrm{Q}}\right), $$

so that

$$ {\mathrm{W}}_{\mathrm{P}\mathrm{Q}\mathrm{S}} = \left(\ {\mathrm{W}}_0\kern0.5em \times \kern0.5em \left({\mathrm{P}}_{\mathrm{HS}} + {\mathrm{P}}_{\mathrm{P}\mathrm{Q}}\right)\ \hbox{-}\ {\mathrm{P}}_{\mathrm{HS}}\times {\mathrm{W}}_{\mathrm{HS}}\right) \div {\mathrm{P}}_{\mathrm{P}\mathrm{Q}} = 3193\mathrm{g}. $$

So, the difference in mean birth weight attributable to the offer of incentives, amongst those women with the potential to benefit, can be estimated as 3338 − 3193 = 145 g.

In order to assess the statistical significance of this difference, we applied a bootstrapping procedure, with 10,000 replicated datasets, giving a 95% confidence interval for this difference of −617 g to +803 g. This confidence interval is wide, and includes zero, so there is no statistical evidence of an increase in birth weight attributable to the intervention.

**Fig. 2:**
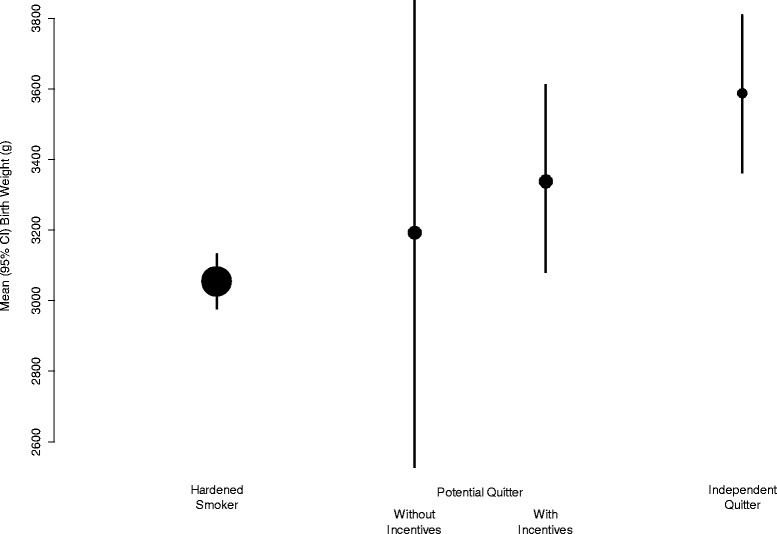
Mean (95% CI) birth weight (in g), in babies born to hardened smokers, independent quitters, and potential quitters (with or without the offer of incentives). Size of *circles* is in proportion to the number of babies

The bootstrapping procedure also provided confidence intervals for the estimates of mean birth weight for each subgroup separately: the hardened smokers, independent quitters, and the potential quitters with incentives (who continued to smoke) or with incentives (who were able to quit), as shown in Fig. 2. There is huge uncertainty in the estimated mean birth weight of the potential quitters who were not offered incentives. Nevertheless, the trend in estimated mean birth weights from the hardened smokers to the independent quitters is striking, and illustrates that the potential quitters are an intermediate group, with average birth weights between those of the two extreme groups.

### Complier average causal effect (CACE) method to uncover an incentives effect hidden by ITT analysis

In the simplest formulation of a CACE analysis, participants are classified as compliers or non-compliers with the intervention. For a continuous outcome, the mean value in the intervention group (μ_1_) and the control group (μ_0_), are viewed as weighted means of the mean values in these two subgroups of participants:

$$ {\upmu}_0 = {\uppi}_{\mathrm{c}\kern0.1em }{\upmu}_{\mathrm{c}0} + {\uppi}_{\mathrm{n}}\kern0.1em {\upmu}_{\mathrm{n}},\ \mathrm{and} $$

$$ {\upmu}_1 = {\uppi}_{\mathrm{c}\kern0.1em }{\upmu}_{\mathrm{c}1}+{\uppi}_{\mathrm{n}\kern0.1em }{\upmu}_{\mathrm{n}} $$

where π_c_ and π_n_ are the proportions of compliant and non-compliant individuals in the population, μ_n_ is the mean response for non-compliant individuals (which is the same in the intervention and control groups), and μ_c0_ and μ_c1_ are the mean outcomes for compliant individuals, which depends on whether the individual is randomised to the intervention or control group (and therefore receives the intervention or not). Under this formulation, the CACE can be estimated as:

$$ {\upmu}_{\mathrm{c}1}\hbox{-}\ {\upmu}_{\mathrm{c}0}\kern0.5em =\kern0.5em \left({\upmu}_1\hbox{-}\ {\upmu}_0\right)\kern0.5em \div \kern0.5em {\uppi}_{\mathrm{c}} $$

However, in this situation, all women in the intervention group were offered incentives to stop smoking, i.e. all intervention group women “received” the intervention, and were therefore compliant with it. On the face of it, a CACE analysis in this situation is not applicable.

The above description of the CACE analysis is actually a simplification of a more general formulation. In general, the population can be divided into four groups: compliers (who receive the intervention if it is offered), non-compliers (who do not receive the intervention when it is offered), “always takers” (who receive the intervention whether it is offered or not), and “defiers” (who receive the intervention when it is not offered, and do not receive it when it is offered). The simple formulation described above is based on the (not unreasonable) assumption that in a randomised trial, there is no opportunity to be an always taker or a defier (i.e. π_a_ and π_d_ = 0). However, the general CACE formulation begins to make sense when we see that it is an instrumental variable analysis: in general, the CACE analysis treats randomisation to the intervention as an instrumental variable, which can only affect the outcome via the mediator (receiving the intervention). The language used in the CACE formulation maybe does not fit, but the principles do: in our data, we assume that the instrumental variable of randomisation to receive the offer of incentives to stop smoking can only affect birth weight via the mediator of quitting smoking. “Compliers” are now seen as women who quit when randomised to be offered incentives, but continue to smoke when randomised to not receive the offer of incentives, “non-compliers” are those who continue to smoke whether randomised to be offered incentives or not, and “always takers” are women who quit regardless of which group they are randomised into. We can assume that there are no “defiers” (i.e. women who quit only when given no incentive, but refuse to quit if offered incentives).

The mean outcome in the two groups can now be seen as:

$$ {\upmu}_0\kern0.5em =\kern0.5em {\uppi}_{\mathrm{c}\kern0.5em }{\upmu}_{\mathrm{c}0}\kern0.5em  + {\uppi}_{\mathrm{n}}\kern0.5em {\upmu}_{\mathrm{n}} + {\uppi}_{\mathrm{a}}\kern0.5em {\upmu}_{\mathrm{a}\kern0.5em }\mathrm{and} $$

$$ {\upmu}_1\kern0.5em =\kern0.5em {\uppi}_{\mathrm{c}\kern0.5em }{\upmu}_{\mathrm{c}1} + {\uppi}_{\mathrm{n}}\kern0.5em {\upmu}_{\mathrm{n}} + {\uppi}_{\mathrm{a}}\kern0.5em {\upmu}_{\mathrm{a}} $$

We assume that the average outcome for those who never quit or always quit are the same regardless of the randomisation. So, as in the simple setting, we can estimate the difference in outcome between compliers in the two randomised groups is:

$$ {\upmu}_{\mathrm{c}1}\kern0.5em \hbox{-} \kern0.5em {\upmu}_{\mathrm{c}0} = \left(\ {\upmu}_1\kern0.5em \hbox{-}\ {\upmu}_0\right) \div {\uppi}_{\mathrm{c}} $$

Therefore, the CACE analysis simplifies to the average birth weight of babies of intervention participants (3141 g) minus the average birth weight of control participants (3120 g) divided by the proportion of women who stop smoking only when offered an incentive. Since the total proportion who quit smoking in the incentives group is π_a_ + π_c_, and in the no incentives group is π_a_, we can estimate π_c_ as the difference in the proportion who quit smoking between the two randomised groups (0.143), i.e.:

$$ {\upmu}_{\mathrm{c}1}\hbox{-}\ {\upmu}_{\mathrm{c}0}\kern0.5em  = \left(3141\mathrm{g}\ \hbox{--}\ 3120\mathrm{g}\right) \div \kern0.5em 0.143 = 145\mathrm{g} $$

This is exactly the same value as was obtained by the intuitive approach described earlier. In fact, the two approaches are the same; the group we identified as “independent quitters” are the “always takers” of CACE notation, the “hardened smokers” are “non-compliers”, and “potential quitters” are “compliers”.

## Discussion

The estimated increase in birth weight caused by the offer of incentives in the CPIT trial was 21 g overall, but for the subgroup of women who quit as a result of the offer of incentives, and would not have been able to do so without the offer, it was 145 g. Throughout, we have viewed this as the causal effect of the intervention, within the subgroup who are susceptible to (comply with) it, but the value of 145 g can equally be seen as the causal effect of stopping smoking on birth weight, within this subgroup of women for whom the offer of incentives is sufficient to help them to quit. This is in line with birth weight differences associated with smoking cessation recorded by other researchers - 160 grams for light smoking (1–9 cigarettes per day) and 230 grams for heavy smoking (>9 cigarettes per day) [8].

To try to understand this analysis, Fig. 1 gives the raw data. Comparison of those who continued to smoke shows that those in the incentives group had babies with a slightly lower birth weight on average compared with those who continue to smoke in the control group. This indicates that the other variables related to birth weight (that make up the 450 g difference in birth weight between continuing smokers and quitters) were more prevalent in those who were unable to quit in the intervention group. Comparison of those who quit shows that those in the control group were all independent quitters who had other characteristics associated with higher birth weight, whereas some in the intervention group are potential quitters who are likely to have other characteristics that reduce birth weight. Overall comparison of those who continued to smoke and those who quit from both groups shows a 450 g difference in birth weight. Our analysis indicates that only 145 g of the 450 g difference in birth weight between continuing smokers and quitters is due to smoking cessation that takes place after maternity booking. The last comparison shows that overall the offer of incentives to pregnant smokers only increases birth weight by 21 g on average compared with those not offered incentives.

Figure 2 illustrates the birth weight of babies born to hardened smokers (those who continued to smoke even when offered incentives to quit - calculated from the intervention group) was 3050 g and the birth weight of babies born to those who independently quit without the offer of incentives (those who quit without incentives - calculated from the control group quitters) was 3600 g. Potential quitters from each group are calculated by removing the above groups’ numbers and weights from the overall birth weight of babies in each group and then dividing the overall remaining birth weight of babies in each group by the number of potential quitters (14.3%) for each group (those who quit because of the offer of incentive payments). These calculated values are for potential quitters in each group (in the incentives group women who quit because they were offered incentive payments, in the control group those who did not quit because they were not offered incentive payments). The difference between these two calculated values is 145 g and represents the difference in birth weight caused by the incentive payments offered to quit smoking. The other differences between women are responsible for the further 400 g difference in birth weight between babies born to hardened smokers and independent quitters. These differences may include both characteristics related to smoking such as number of cigarettes smoked per day and partner smoking and other characteristics not directly related to smoking such as maternal deprivation score (as illustrated in Additional file 3: Table S2 reference [11]).

We initially thought that a CACE analysis was not relevant, since the notion of compliance and non-compliance (e.g. taking or not taking tablets in a drug trial) was not applicable in a trial of the offer of incentives, which was received by all women randomised to receive it (and none of those randomised to not receive it). We therefore pursued an intuitive analysis, estimating the mean birth weight of two subgroups of women unaffected by the intervention (hardened smokers and independent quitters), leading to estimates of the mean birth weight for a third group of potential quitters, with and without the offer, and therefore to the estimate of the causal effect of the intervention for these women.

We found, however, that the CACE approach could be used, even though the terminology was slightly incongruous in this setting. This gave us some reassurance that our intuitive approach was valid. Also, whilst the CACE approach gave an estimate of the causal effect of the intervention, the intuitive approach provided us with estimates of the mean birth weight of each subgroup separately, and for the potential quitters, of the mean birth weight with and without the offer of incentives. We feel that this has given us a greater understanding of the characteristics of these subgroups and the impact of the intervention.

It should be noted that: (1) the trial aimed to examine the effect of the offer of financial incentives in addition to routine smoking cessation service support to engage with smoking cessation services and/or quit smoking during pregnancy. This means that examining the effect on birth weight only in potential quitters (those who can quit only with the offer of incentives) may be of limited public health importance; (2) neither of these estimates is anywhere near statistically significant (95% CI −615 g to +803 g). In order for a trial to have 80% power to detect an effect on birth weight, if we take 100 g as a clinically important effect (in those who manage to quit as a result of the intervention), then we estimate a study of 27,637 participants (per group, or 55,274 in total) would be required. This assumes that 14.3% of women stop smoking as a result of the offer of incentives, so the mean difference in birth weight between randomised groups will be 14.3 g. This is clearly not feasible. In order to improve the power of a trial to detect an effect on birth weight, then the intervention itself must be improved to increase the impact on quit rates (e.g. by optimising the package of incentives on offer), the intervention must be targeted at those most likely to be a potential quitter (which would likely be seen as unethical), or the analyses outlined in this report must be improved to estimate the causal effect of the intervention on birth weight.

Since completing the analyses described in this report, we have become aware of two avenues for further work. Our analyses are the same as a very simple two-stage least squares analysis [15]; we confirmed this using the AER package within R [16] and obtained the same estimate of the birth weight difference due to the intervention, 145.2 g, with a 95% CI of −511.4 g to 801.7 g, which is not dissimilar to our CI, derived by bootstrapping. Another way the data could be analysed is as a mixture model within a Bayesian framework [17]. Each of these methods could be used to develop more complex models incorporating covariate effects, potentially reducing the uncertainty around the estimate of the effect of the intervention on birth weight. It might therefore be possible to design a study powered to detect such effects, without the need for tens of thousands of randomised participants.

Nevertheless, whilst an overall 21-g average improvement in birth weight may appear trivial, our analyses show that for individual mothers who benefit from the intervention and manage to give up smoking during pregnancy as a result, there may be a substantial increase in the birth weight of their infant.

### Limitations of the study

This study uses secondary outcome data from a randomised controlled trial. It adds to evidence from trial data on smoking cessation in pregnancy in relation to improvements in birth weight [9]. Our analysis demonstrates how the CACE framework can be used to estimate the causal impact of an intervention to improve smoking quit rates, on a “downstream” outcome, birth weight. By approaching the same problem from an intuitive angle, we also derived estimates of mean birth weight in two subgroups of women who are not directly observed. We have not adjusted our analyses for any baseline characteristics; if this is possible, it may provide more precise estimates of the causal effect of the intervention on birth weight, and ultimately make possible a large-scale trial, powered to detect such effects. We shall continue to investigate this possibility.

### Implications for practice

This analysis supports the offer of financial incentives to help pregnant smokers to stop smoking during pregnancy in order to improve outcomes for infants, by increasing their birth weight.

## Conclusion

In the current financial climate, advocating the offer of financial incentives to pregnant smokers to engage with smoking cessation services and or quit smoking is difficult. Demonstrating that the clinically insignificant improvement in average birth weight of 21 g hides a clinically significant estimated increase in birth weight of 145 g, amongst those women who manage to give up smoking as a result of the intervention, helps to justify the offer of incentives for smoking cessation as a reasonable intervention to consider.

## Additional files

Additional file 1: Flow diagram of the CPIT II [11]. (DOCX 2446 kb)

Additional file 2: Consort checklist submitted with the manuscript for the CPIT II [11]. (DOC 219 kb)

Additional file 3: Table S2. from the CPIT [11] (DOCX 13 kb)

## Abbreviations

CACE: complier average causal effects
CPIT: Cessation in Pregnancy Incentives Trial
NHS: National Health Service
