# Supplementary material for: Birth weight differences between those offered financial voucher incentives for verified smoking cessation and control participants enrolled in the Cessation in Pregnancy Incentives Trial (CPIT), employing an intuitive approach and a Complier Average Causal Effects (CACE) analysis
Source: Trials. 2017 Jul 20;18:337. doi: 10.1186/s13063-017-2053-x (PMC5520300; doi:10.1186/s13063-017-2053-x)
Supplement: Supplementary file 3 — from the CPIT [11] (DOCX 13 kb) [file 13063_2017_2053_MOESM3_ESM.docx]

**Table S2 Baseline characteristics of trial population by group allocation**

| **Characteristics** | **Control group** | |  | **Incentives group** | |  |
| --- | --- | --- | --- | --- | --- | --- |
|  | **Participants (n=303)*** | **No with missing data** |  | **Participants (n=306)** | **No with missing data** |  |
| **Routinely collected maternity booking data** | | | | | | |
| Mean (SD) height (m) | 1.63 (0.06) | 2 |  | 1.62 (0.07) | 8 |  |
| Mean (SD) weight (kg) | 69.37 (16.12) | 2 |  | 69.82 (16.34) | 8 |  |
| Mean (SD) body mass index (kg/m^2^) | 26.23 (5.89) | 2 |  | 26.40 (5.77) | 8 |  |
| White ethnicity | 98.7 | 0 |  | 99.7 | 0 |  |
| Mean (SD) maternal age at estimated delivery (years) | 27.66 (6.07) | 0 |  | 28.27 (5.77) | 0 |  |
| Mean (SD) gestation at booking (weeks) | 12.62 (2.68) | 0 |  | 12.27 (2.46) | 0 |  |
| **From routine dataset collected by health board** | | | | | | |
| Median (range) previous live births | 1 (0-6) | 9 |  | 1 (0-6) | 8 |  |
| Median (range) previous stillbirth or miscarriage | 0 (0-7) | 50 |  | 0 (0-10) | 37 |  |
| **Routinely collected maternity booking data** | | | | | | |
| SIMD fifth†: |  |  |  |  |  |  |
| 1st fifth (most deprived) | 65.0 | 0 |  | 67.0 | 0 |  |
| 2nd fifth | 17.8 | 0 |  | 16.3 | 0 |  |
| 3rd fifth | 10.9 | 0 |  | 9.2 | 0 |  |
| 4th fifth | 3.6 | 0 |  | 3.9 | 0 |  |
| 5th fifth (least deprived) | 2.6 | 0 |  | 3.6 | 0 |  |
| Mean (SD) baseline carbon monoxide (ppm) | 13.65 (6.34) | 0 |  | 13.14 (6.41) | 0 |  |
| Fagerstrom questionnaire (smoking history variables): |  |  |  |  |  |  |
| First cigarette within 5 mins of waking | 57.1 | 0 |  | 52.9 | 0 |  |
| Difficulty not smoking | 31.4 | 0 |  | 27.1 | 0 |  |
| 1st cigarette most difficult to give up | 65.3 | 0 |  | 59.8 | 0 |  |
| Smoke >20/day | 17.1 | 0 |  | 10.5 | 0 |  |
| Smoke more in morning | 55.1 | 0 |  | 48.0 | 0 |  |
| Smoke when ill | 59.7 | 0 |  | 48.7 | 0 |  |
| Partner smokes | 66.3 | 0 |  | 59.8 | 0 |  |
| Mean (SD) Fagerstrom score‡ | 5.32 (2.21) | 0 |  | 4.85 (2.22) | 0 |  |

SIMD=Scottish index of multiple deprivation.

*****Consent withdrawn post-randomisation for three women.

†Derived from postcode of residence.

‡Uses first six smoking history variables from Fagerstrom questionnaire. Score of ≥5 indicates significant nicotine dependence.
