# Supplementary material for: Birth weight differences between those offered financial voucher incentives for verified smoking cessation and control participants enrolled in the Cessation in Pregnancy Incentives Trial (CPIT), employing an intuitive approach and a Complier Average Causal Effects (CACE) analysis
Source: Trials. 2017 Jul 20;18:337. doi: 10.1186/s13063-017-2053-x (PMC5520300; doi:10.1186/s13063-017-2053-x)
Supplement: Supplementary file 3 [file 13063_2017_2053_MOESM1_ESM.docx]

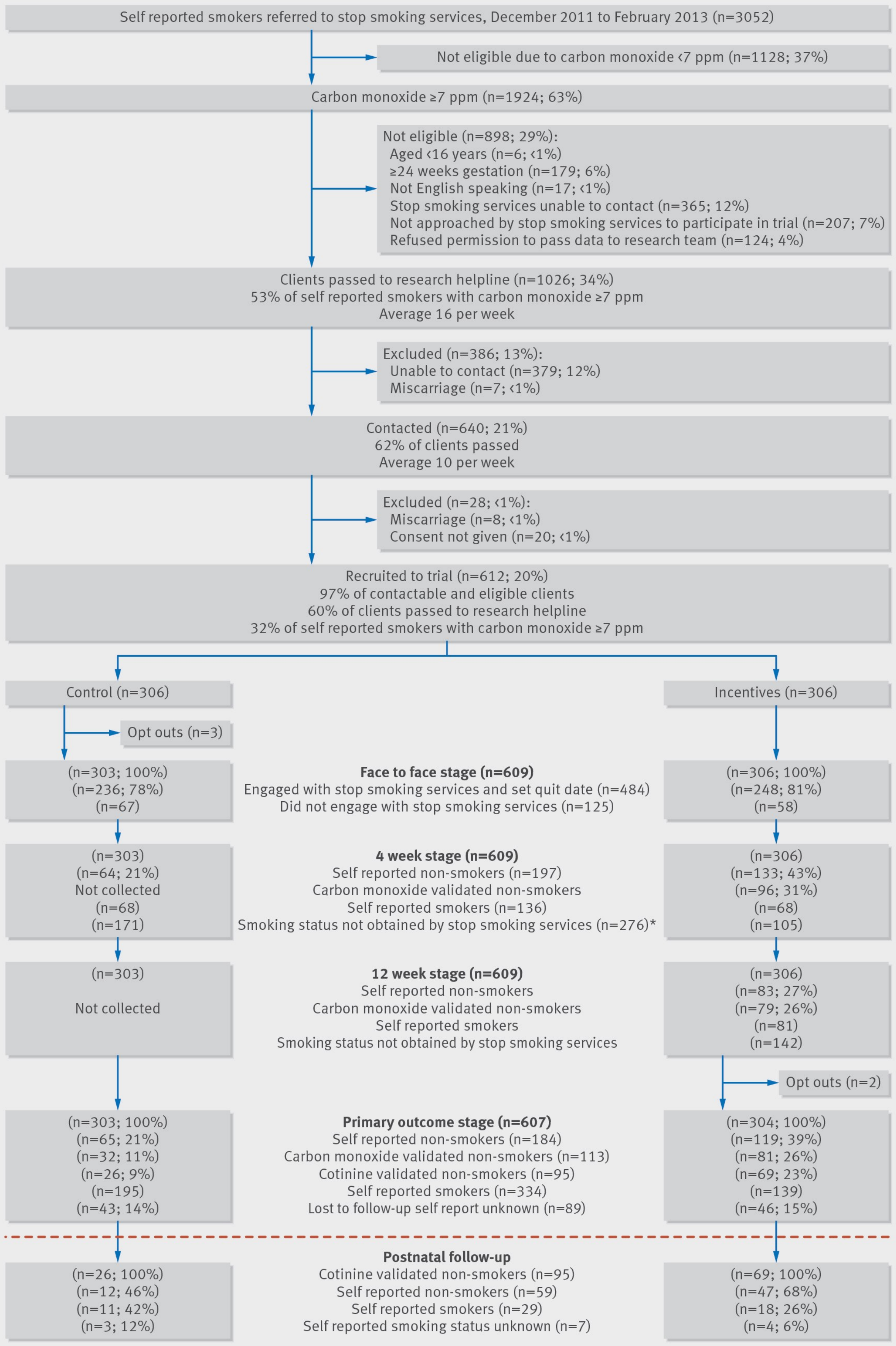

* Includes participants who did not engage with stop smoking services

† Includes participants who were not carbon monoxide validated non-smokers at 4 weeks
